# *SLC7A8* overexpression inhibits the growth and metastasis of lung adenocarcinoma and is correlated with a dismal prognosis

**DOI:** 10.18632/aging.205446

**Published:** 2024-01-18

**Authors:** Fang-Ming Wang, Li-Qiang Xu, Zhong-Chao Zhang, Qiang Guo, Zhi-Peng Du, Yue Lei, Xu Han, Chuang-Yan Wu, Feng Zhao, Jiu-Ling Chen

**Affiliations:** 1 Department of Thoracic Surgery, Union Hospital, Tongji Medical College, Huazhong University of Science and Technology, Wuhan, China; 2 Department of Cardiothoracic Surgery, Taihe Hospital, Hubei University of Medicine, Shiyan, China; 3 Department of Gastroenterology, Institute of Liver and Gastrointestinal Diseases, Tongji Hospital, Tongji Medical College, Huazhong University of Science and Technology, Wuhan, China; 4 Department of Blood Transfusion, Taihe Hospital, Hubei University of Medicine, Shiyan, China

**Keywords:** *SLC7A8*, lung adenocarcinoma, prognosis, TCGA, immune infiltration

## Abstract

Background: Overexpression of solute carrier family 7 member 8 (SLC7A8) has been shown to relate to the survival time and tumor progression in cancer patients. However, the role of *SLC7A8* in lung adenocarcinoma (LUAD) is still obscure.

Method: The relationships between *SLC7A8* expression in LUAD tissues and clinical values as well as immune infiltration were explored through bioinformatics. The functions and pathways of *SLC7A8* in LUAD were investigated using Kyoto Encyclopedia of Genes and Genomes enrichment analysis, Gene Set Enrichment Analysis, Western blotting, and other methods.

Results: We found that the expression of *SLC7A8* was decreased significantly in LUAD tissues compared with normal tissues, which was related to the dismal survival time and disease progression. Moreover, it carried diagnostic value in LUAD and was a risk factor for dismal prognosis. Receiver operating characteristic curve analysis indicated that the expression level of *SLC7A8* carried significant diagnostic value in LUAD. Overexpression of *SLC7A8* inhibited the proliferation, invasion, and migration of LUAD cells, likely through a mechanism involving the cell cycle. *SLC7A8* expression in LUAD was significantly correlated with the infiltration of immune cells, especially B cells, interstitial dendritic cells, mast cells, CD56 bright cells, natural killer cells, plasmacytoid dendritic cells, T follicular helper cells, T helper 2 and 17 cells, and immune factors.

Conclusion: The downregulation of *SLC7A8* was related to a dismal prognosis and immune cell infiltration in LUAD. Increasing the expression of *SLC7A8* inhibited the growth and migration of LUAD cells, thereby improving the prognosis of patients.

## INTRODUCTION

Lung cancer has become one of the leading causes of mortality in the world [1]. Of its different pathological subtypes, non-small cell lung cancer (NSCLC) accounts for about 85% of all lung cancer cases, and lung adenocarcinoma (LUAD) constitutes the highest proportion of NSCLC cases [2]. The relationship between the expression of certain genes and LUAD progression has been increasingly investigated [3–5]. For example, the decreased expression of *TNFAIP3*, an anti-inflammatory gene that helps maintain the inflammatory balance, reduced the proportion of CD8^+^ T cells, thus assisting LUAD cells in immune evasion. Moreover, it caused anti-programmed cell death protein 1 monotherapy to be ineffective and led to poor prognosis in LUAD patients [5].

The expression level of *SLC7A8*, which encodes large neutral amino acid transporter small subunit 2, was shown to be related to tumor progression [6–9]. *SLC7A8* was highly expressed in estrogen receptor (ER)-positive breast cancer and was a marker of good prognosis in these patients [6]. In uterine leiomyoma tissues, luteinizing hormone upregulated *SLC7A8*. Knockdown of *SLC7A8* enhanced the proliferation of uterine leiomyoma cells [7]. A mutation in *SLC7A8* reduced the efficacy of the combination of cisplatin and verapamil for treating esophageal squamous carcinoma and induced the development of drug resistance [8]. Asada et al. showed that *SLC7A8* overexpression improved the survival rate of LUAD patients [9]. However, the role of *SLC7A8* in LUAD progression is still obscure.

This study aimed to further elucidate the relationship between *SLC7A8* expression and LUAD prognostic factors using bioinformatics and cell models. We also examined the role of *SLC7A8* in LUAD cell growth and metastasis *in vitro*.

## RESULTS

### The expression of *SLC7A8* was attenuated in LUAD

**Figure 1:**
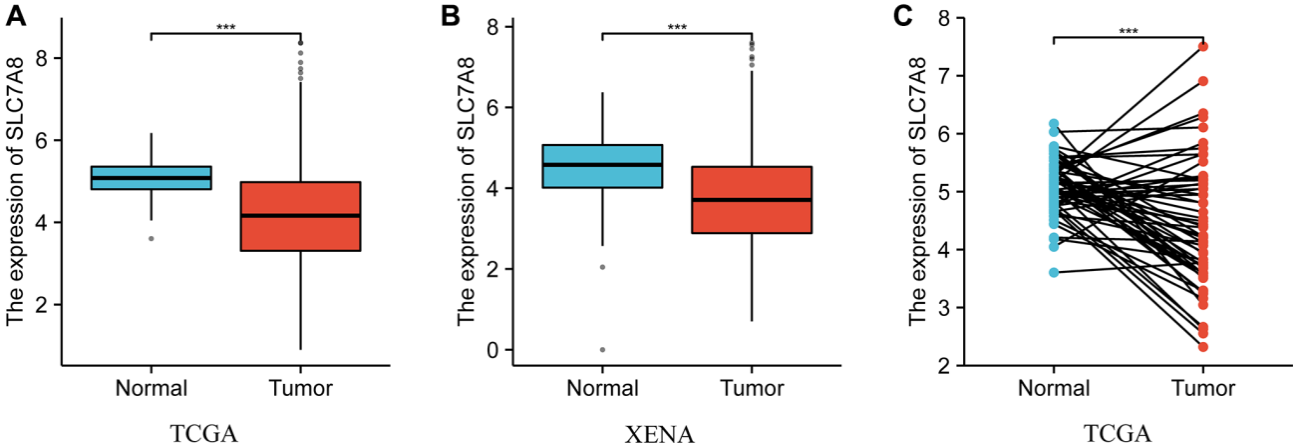
**The expression level of *SLC7A8* in LUAD based on TPM data from TCGA and Xena.** (**A**) TPM data of unpaired tissues from TCGA. (**B**) TPM data of unpaired tissues from Xena. (**C**) TPM data of paired tissues from TCGA. Abbreviations: LUAD: lung adenocarcinoma; TCGA: The Cancer Genome Atlas; TPM: transcripts per million.

The transcripts per million (TPM) data retrieved from The Cancer Genome Atlas (TCGA) and Xena showed that *SLC7A8* was downregulated in LUAD tissues compared with normal lung tissues (Figure 1). The expression of *SLC7A8* was lower in unpaired LUAD tissues than in normal tissues from TCGA and Xena (Figure 1A, 1B) and in paired LUAD tissues from TCGA (Figure 1C).

### Diagnostic value of *SLC7A8* in LUAD

**Figure 2:**
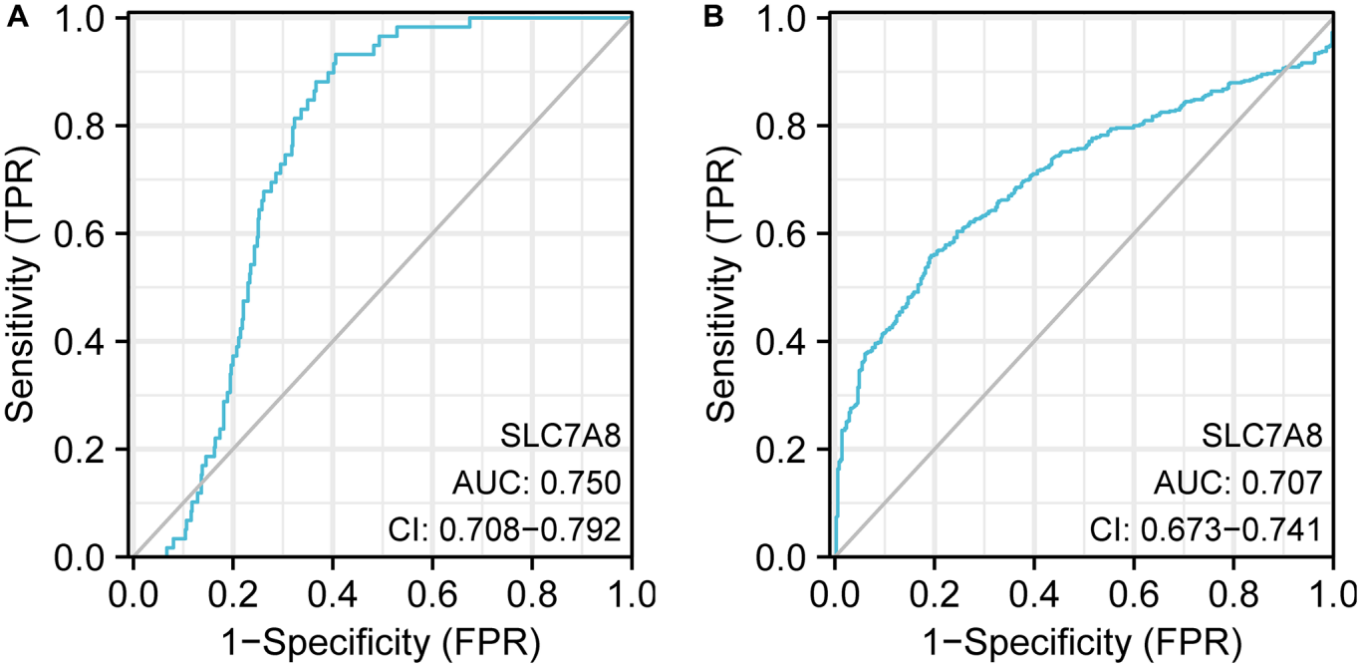
**ROC curve analysis indicating the diagnostic value of *SLC7A8* in LUAD.** (**A**) TPM data from TCGA. (**B**) TPM data from Xena. Abbreviation: ROC: receiver operating characteristic.

Receiver operating characteristic (ROC) curve analysis indicated that the expression level of *SLC7A8* carried significant diagnostic value in LUAD. The area under the curve values of ROC curves plotted based on *SLC7A8* expression data from TCGA and Xena were 0.750 and 0.707, respectively (Figure 2A, 2B), highlighting the important diagnostic value of *SLC7A8* in LUAD.

### Low expression of *SLC7A8* was associated with poor prognosis in LUAD patients

**Figure 3:**
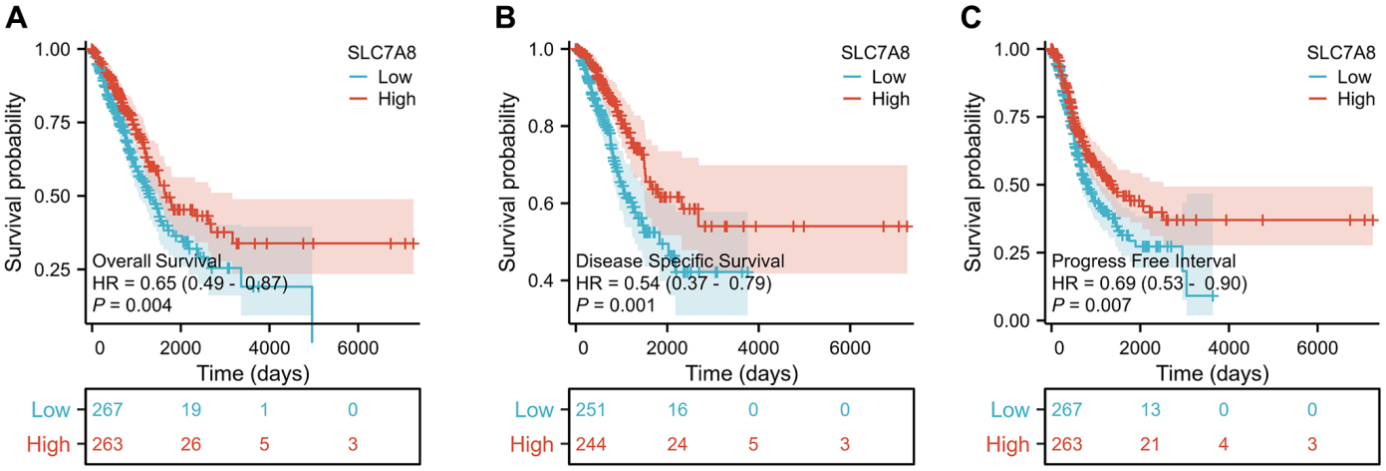
**Expression level of *SLC7A8* is associated with the prognosis of LUAD patients.*** SLC7A8* expression is associated with (**A**) OS in LUAD patients, (**B**) DSS in LUAD patients, (**C**) PFI in LUAD patients. Abbreviations: OS: overall survival; DSS: disease-specific survival; PFI: progression-free interval.

**Figure 4:**
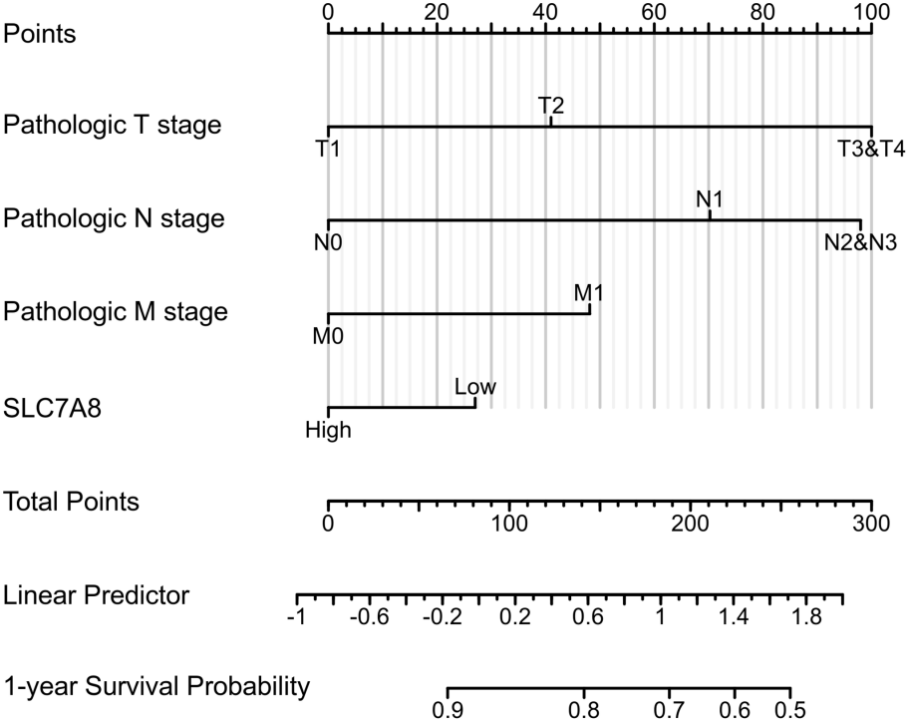
The nomogram shows the correlation between low expression of *SLC7A8* and poor prognosis of LUAD.

Prognostic data based on the TPM values from TCGA revealed that low expression of *SLC7A8* was associated with poor prognosis in LUAD patients, especially with shorter overall survival (OS), disease-specific survival (DSS), and progression-free interval (PFI) (Figure 3A–3C). Cox regression analysis (Table 1) confirmed the association between low *SLC7A8* expression and poor patient prognosis. A nomogram constructed based on the primary therapy outcome, T stage, N stage, M stage, and *SLC7A8* expression accurately predicted patient prognosis (Figure 4).

**Table 1:** Cox regression analysis reveals the prognostic factors in LUAD.

**Characteristics**	***N***	**HR (95% CI)**	***P*-value**
T stage	527		<0.001
T1	176	Reference	
T2	285	1.507 (1.059–2.146)	0.023
T3 and T4	66	3.095 (1.967–4.868)	<0.001
N stage	514		<0.001
N0	345	Reference	
N1	96	2.293 (1.632–3.221)	<0.001
N2 and N3	73	2.993 (2.057–4.354)	<0.001
M stage	381		0.010
M0	356	Reference	
M1	25	2.176 (1.272–3.722)	0.005
Primary therapy outcome	442		<0.001
PD	71	Reference	
SD	38	0.289 (0.141–0.592)	<0.001
PR	5	0.702 (0.170–2.897)	0.625
CR	328	0.266 (0.185–0.382)	<0.001
Race	472		0.191
Asian	8	Reference	
Black or African American	55	1.911 (0.254–14.382)	0.529
White	409	2.714 (0.380–19.403)	0.320
Gender	530		0.570
Female	283	Reference	
Male	247	1.087 (0.816–1.448)	0.569
Age	520		0.185
≤65	257	Reference	
>65	263	1.216 (0.910–1.625)	0.186
SLC7A8	530		0.004
Low	267	Reference	
High	263	0.655 (0.491–0.874)	0.004

### *SLC7A8* overexpression inhibited the proliferation, migration, and invasion of LUAD cells

**Figure 5:**
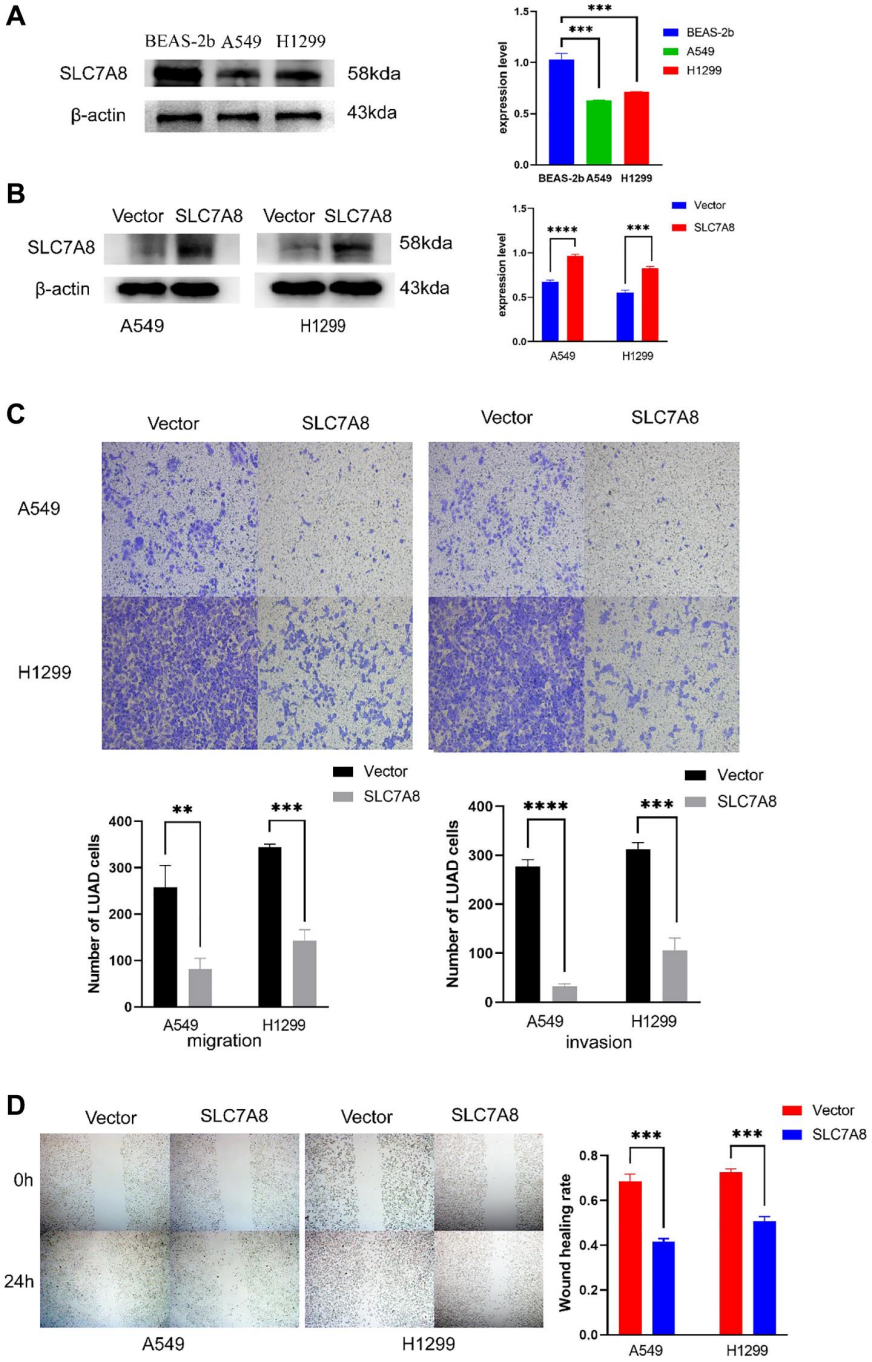
**Overexpression of *SLC7A8* suppresses the migration and invasion of LUAD.** (**A**) The expression level of *SLC7A8* in BEAS-2B, A549, and H1299 cells. (**B**) The protein expression levels were determined by Western blotting. (**C**) The migration and invasion were assessed using Transwell assays. (**D**) The migration ability was determined using wound healing assays as well.

**Figure 6:**
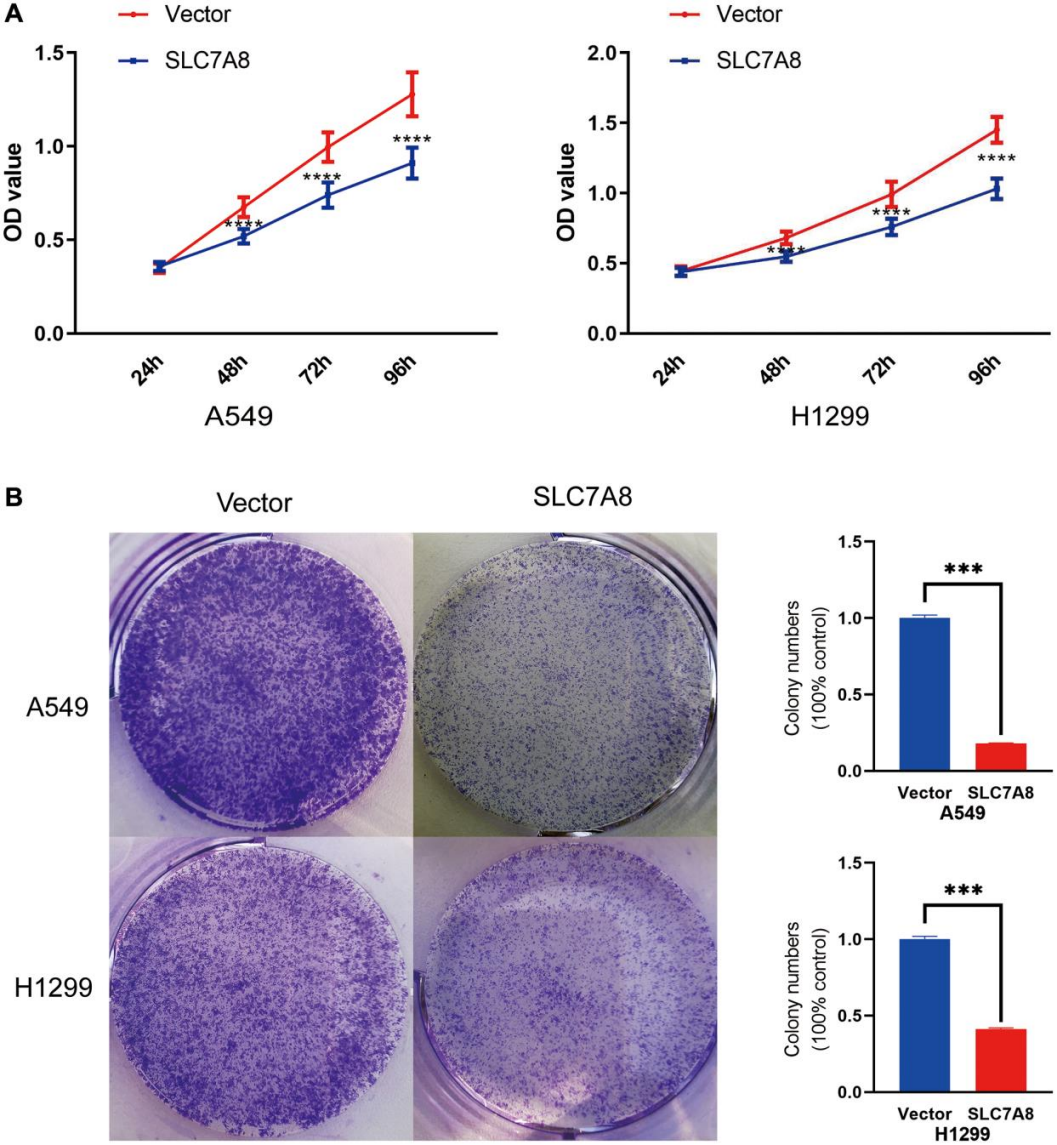
(**A**) Cell proliferation in the *SLC7A8*-overexpressing group was demonstrated using the CCK-8 assays. (**B**) Cell proliferation in the SLC7A8-overexpressing group was demonstrated using and colony forming assays. Abbreviation: CCK-8: Cell Counting Kit-8.

The expression of *SLC7A8* was significantly higher in BEAS-2B cells than in A549 and H1299 cells (Figure 5A). Gene Ontology analysis showed that 679 genes co-expressed with *SLC7A8* were correlated with cell cycle progression (Table 2). The overexpression of SLC7A8 in A549 and H1299 cells was successfully verified using Western blotting (Figure 5B). The Transwell assay showed that the number of A549 and H1299 cells in the *SLC7A8*-overexpression group were significantly lower than in the control group (Figure 5C). Overexpressing *SLC7A8* inhibited the migration of A549 and H1299 cells, as evidenced by the wound healing assay (Figure 5D). The Cell Counting Kit-8 (CCK-8) assay also demonstrated that the number of A549 and H1299 cells significantly decreased in the *SLC7A8*-overexpression group compared with the control group (Figure 6A). The colony forming assay showed that the ability of cells to proliferate was lower in the *SLC7A8*-overexpression group (Figure 6B). These findings indicate that overexpressing *SLC7A8* inhibited the proliferation, migration, and invasion of LUAD cells.

**Table 2:** GO/KEGG analysis of *SLC7A8*.

**Type**	**ID**	**Description**	***P*-value**
BP	GO:0018958	phenol-containing compound metabolic process	6.1974E-06
BP	GO:0030038	contractile actin filament bundle assembly	1.73008E-05
BP	GO:0043149	stress fiber assembly	1.73008E-05
BP	GO:0016999	antibiotic metabolic process	3.44352E-05
BP	GO:0006067	ethanol metabolic process	4.15677E-05
BP	GO:0017000	antibiotic biosynthetic process	4.15677E-05
BP	GO:0031638	zymogen activation	4.78141E-05
BP	GO:0006687	glycosphingolipid metabolic process	5.08109E-05
BP	GO:0006664	glycolipid metabolic process	5.9784E-05
BP	GO:1903509	liposaccharide metabolic process	6.57364E-05
BP	GO:0031032	actomyosin structure organization	9.37131E-05
BP	GO:0006643	membrane lipid metabolic process	0.000154598
BP	GO:0050665	hydrogen peroxide biosynthetic process	0.000160988
BP	GO:0072073	kidney epithelium development	0.00019478
BP	GO:0045056	transcytosis	0.000216961
BP	GO:0001655	urogenital system development	0.000220889
BP	GO:0006665	sphingolipid metabolic process	0.000222584
BP	GO:0072001	renal system development	0.000256287
BP	GO:0061337	cardiac conduction	0.000301821
BP	GO:0051492	regulation of stress fiber assembly	0.000393813
BP	GO:0032355	response to estradiol	0.000438313
BP	GO:0006068	ethanol catabolic process	0.000447416
BP	GO:0072520	seminiferous tubule development	0.000447416
BP	GO:0042403	thyroid hormone metabolic process	0.000475202
BP	GO:0051017	actin filament bundle assembly	0.000487038
BP	GO:0030324	lung development	0.000518227
BP	GO:0071560	cellular response to transforming growth factor beta stimulus	0.000542241
BP	GO:0042445	hormone metabolic process	0.000618613
BP	GO:0061572	actin filament bundle organization	0.000630999
BP	GO:0031670	cellular response to nutrient	0.000650715
BP	GO:0051145	smooth muscle cell differentiation	0.000650715
BP	GO:1901616	organic hydroxy compound catabolic process	0.000650715
BP	GO:0030323	respiratory tube development	0.000659255
BP	GO:0007015	actin filament organization	0.000692946
BP	GO:0071559	response to transforming growth factor beta	0.000724183
BP	GO:0051056	regulation of small GTPase mediated signal transduction	0.000749109
BP	GO:0060541	respiratory system development	0.000789527
BP	GO:0006631	fatty acid metabolic process	0.000820985
BP	GO:0021692	cerebellar Purkinje cell layer morphogenesis	0.000858959
BP	GO:0097202	activation of cysteine-type endopeptidase activity	0.000858959
BP	GO:0043268	positive regulation of potassium ion transport	0.000865894
CC	GO:0042599	lamellar body	0.000150683
CC	GO:0062023	collagen-containing extracellular matrix	0.000312972
KEGG	hsa00280	Valine, leucine and isoleucine degradation	4.78595E-06
KEGG	hsa00380	Tryptophan metabolism	1.1307E-05
KEGG	hsa00340	Histidine metabolism	8.15346E-05
KEGG	hsa05224	Breast cancer	0.000186964
KEGG	hsa00410	beta-Alanine metabolism	0.000512328
KEGG	hsa00071	Fatty acid degradation	0.000757955
KEGG	hsa00600	Sphingolipid metabolism	0.001463422
KEGG	hsa04142	Lysosome	0.00171453
KEGG	hsa05226	Gastric cancer	0.00214208
KEGG	hsa00620	Pyruvate metabolism	0.002161182
KEGG	hsa04115	p53 signaling pathway	0.003838081
KEGG	hsa04110	Cell cycle	0.004060117

Abbreviations: GO: Gene Ontology; KEGG: Kyoto Encyclopedia of Genes and Genomes.

### Mechanisms and protein–protein interaction (PPI) network of SLC7A8

**Figure 7:**
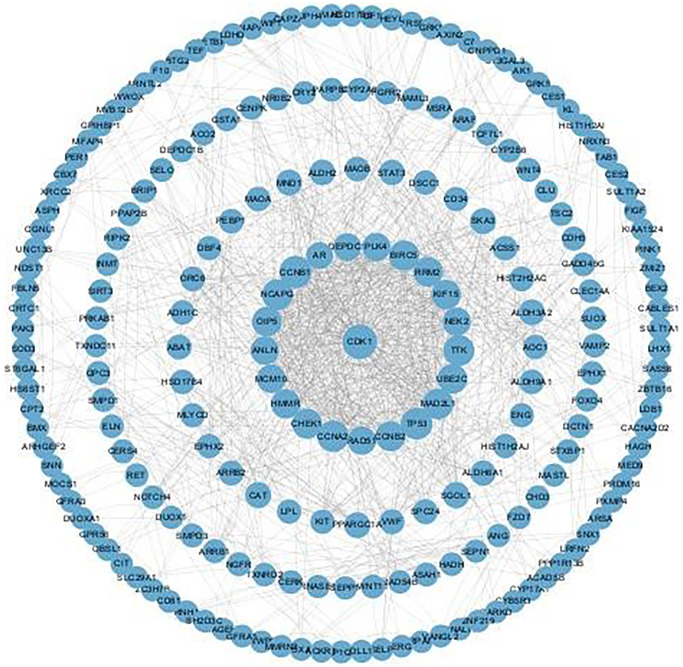
**PPI network of genes strongly co-expressed with *SLC7A8*.** Abbreviation: PPI: protein–protein interaction.

**Figure 8:**
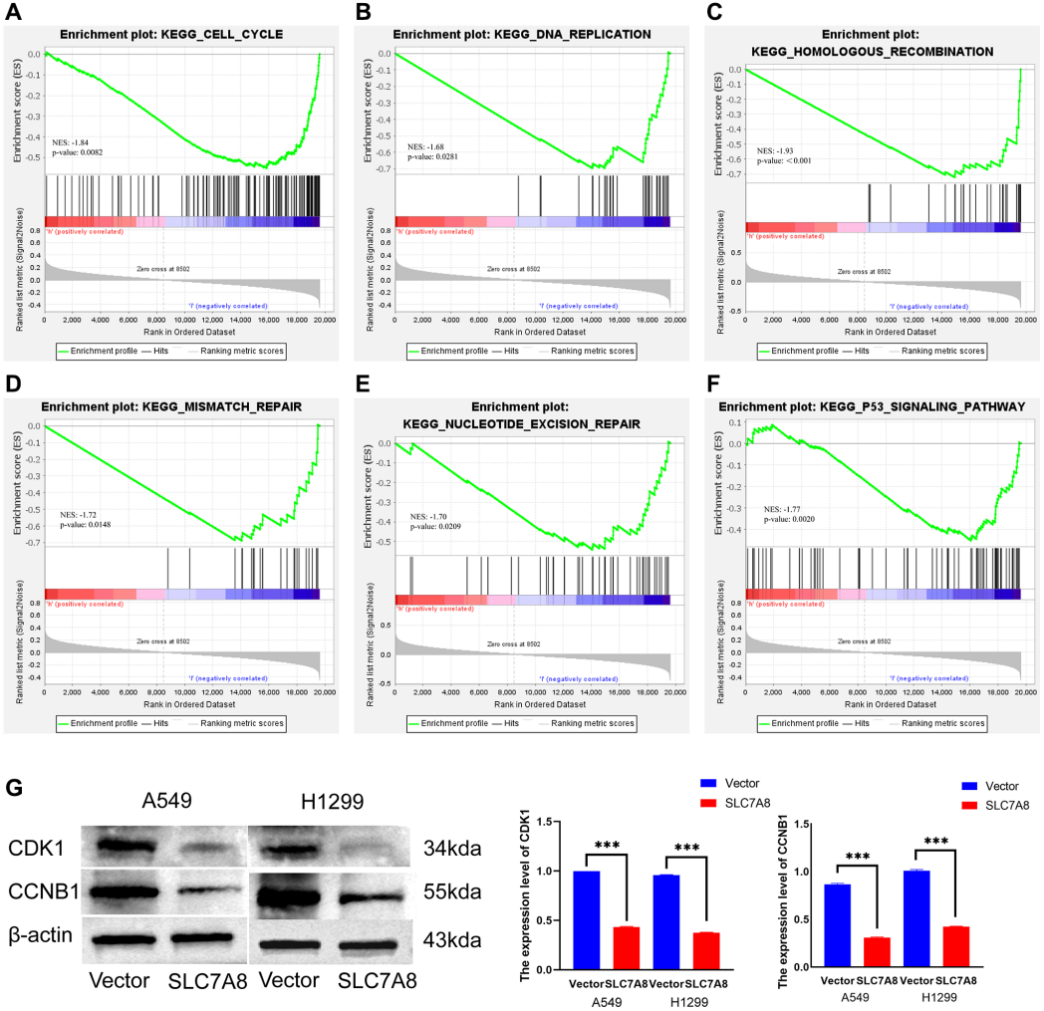
**Mechanisms associated with *SLC7A8*.** (**A**–**F**) Mechanisms associated with *SLC7A8* were explored using GSEA. (**G**) The expression levels of the cell cycle proteins CDK1 and CCNB1. Abbreviations: GSEA: Gene Set Enrichment Analysis; CDK1: cyclin-dependent kinase-1; CCNB1: cyclin B1.

The PPI network shows protein relationships among *SLC7A8*-related genes (Figure 7). Kyoto Encyclopedia of Genes and Genomes enrichment analysis and Gene Set Enrichment Analysis (GSEA) indicated that SLC7A8 influenced the cell cycle and DNA replication (Figure 8A–8F and Table 2). We conducted relevant experiments to validate these preliminary findings and found that the expression of cyclin-dependent kinase-1 (CDK1) and cyclin B1 (CCNB1), two important cell cycle proteins, was significantly attenuated in LUAD cells overexpressing *SLC7A8* (Figure 8G). These results suggest that overexpressing *SLC7A8* may potentially hinder the progression of LUAD by inhibiting the cell cycle pathway.

### *SLC7A8* expression was correlated with infiltrated immune cells and immune factors in LUAD

**Figure 9:**
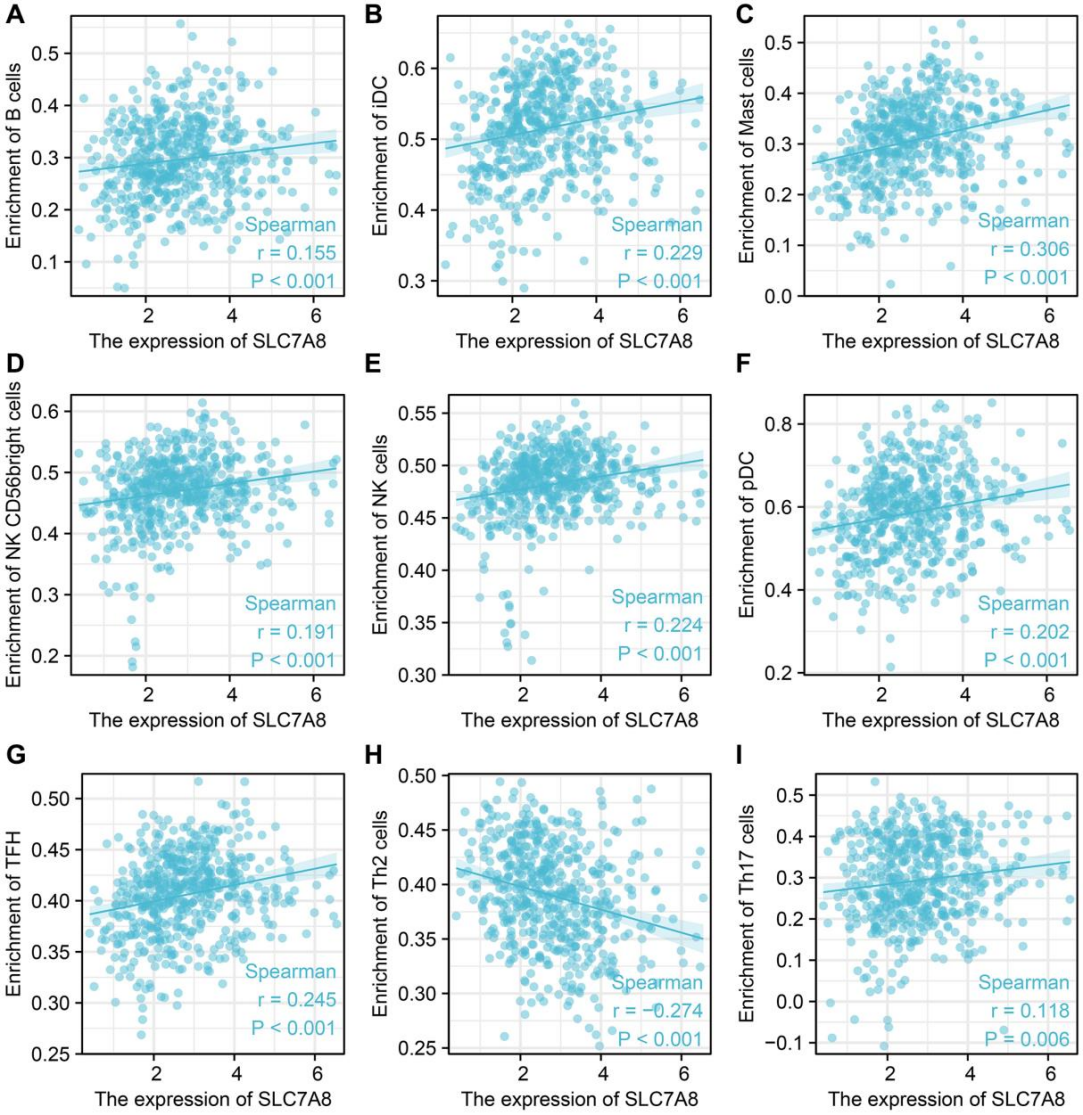
***SLC7A8* expression is correlated with immune infiltrated cells.** (**A**) B cells. (**B**) iDC. (**C**) Mast cells. (**D**) NK CD56 bright cells. (**E**) NK cells. (**F**) pDC. (**G**) TFH. (**H**) Th2 cells. (**I**) Th17 cells.

**Figure 10:**
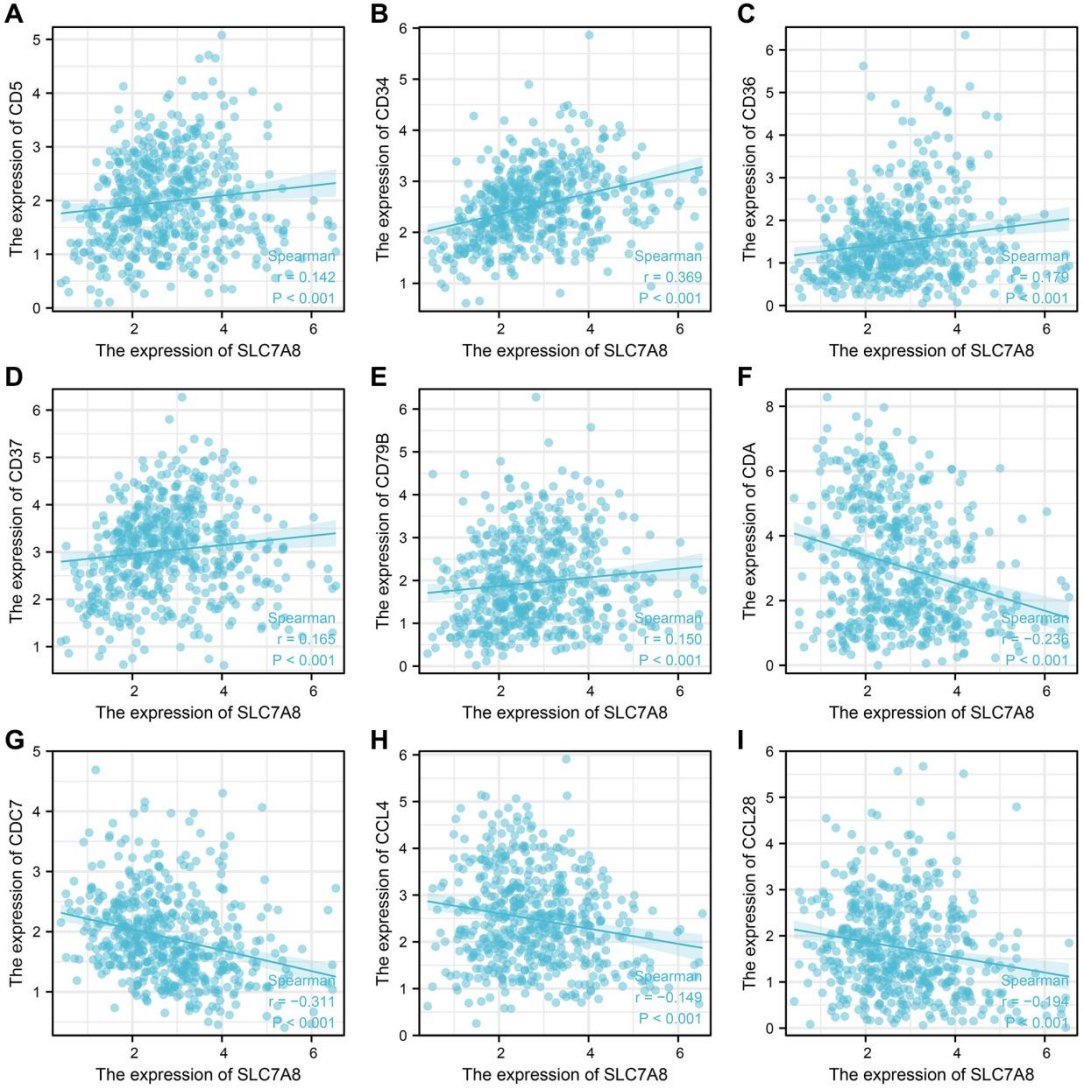
***SLC7A8* expression is correlated with immune factors.** (**A**) CD5. (**B**) CD34. (**C**) CD36. (**D**) CD37. (**E**) CD79B. (**F**) CDA. (**G**) CDC7. (**H**) CCL4. (**I**) CCL28.

The expression level of *SLC7A8* was significantly correlated with the level of immune cell infiltration in LUAD, especially that of B cells, interstitial dendritic cells, mast cells, CD56 bright cells, natural killer cells, plasmacytoid DCs, T follicular helper cells, T helper 2 cells, T helper17 cells (Figure 9). *SLC7A8* expression was also associated with some immune factors, such as CD5, CD34, CD36, CD37, CD79B, CDA, CDC7, CCL4, and CCL28 (Figure 10).

## DISCUSSION

Although progress in the treatment and early screening of lung cancer have mitigated its incidence and mortality, it still accounts for a high percentage of cancer-related deaths [10]. Studies have identified some genes associated with cancer progression. *SLC7* genes were reported to be correlated with the prognosis of lung cancer [11–14]. *SLC7A5* was overexpressed in LUAD and significantly correlated with the Ki-67 labeling index, which can increase metabolic activity and is associated with tumor cell growth [11]. *SLC7A8* was shown to be a good prognostic marker for breast cancer. When overexpressed, it behaved like a tumor suppressor, especially in low-growth ER-positive subtypes [6]. Jiang et al. found that SLC7A2 increased drug sensitivity, immune infiltration, and survival in NSCLC [12]. The upregulation of *SLC7A7* increased the levels of arginine in tumor cells, thus promoting their migration and invasion [13]. Conversely, knockdown of *SLC7A9* inhibited the migration, invasion, and proliferation of esophageal squamous cell carcinoma cells, making it a suitable biomarker to predict lymph node metastasis of this cancer [14]. Our studies showed that *SLC7A8* was lowly expressed in LUAD, whereas overexpression of *SLC7A8* was associated with higher OS, DSS, and PFI, making it a potential prognostic factor for LUAD.

The immune microenvironment intimately affects the progression of LUAD [15–17]. Cancer-related immune cells were shown to serve as biomarkers for the progression of NSCLC [18]. Genes from the *SLC7* family, such as *SLC7A11*, were shown to be associated with the immune microenvironment [19]. B cells, which are involved in humoral immunity, were shown to be an important component of the immune microenvironment of lung cancer. Moreover, differentiated plasma cells were shown to produce antibodies against tumor antigens, which contributed to the immune protection in lung cancer patients [20]. As an example of cell-mediated immunity, Th17 cells produced interleukin-22, which promoted the growth, migration, and invasion of tumor cells [21, 22]. Interleukin-22 also induced tumor cells to express CD155, which inhibits the function of NK cells and supports tumor cell metastasis [23]. In this study, we found that *SLC7A8* expression was associated with B cells, interstitial DCs, mast cells, CD56 bright cells, NK cells, plasmacytoid DCs, T follicular helper cells, Th2 cells, Th17 cells. Moreover, it was correlated with several immune factors, including CD5, CD34, CD36, CD37, CD79B, CDA, CDC7, CCL4, and CCL28. These results suggest that immune factors were significant in deciding the prognostic value of *SLC7A8* in LUAD. We believe that elucidating the relationship between *SLC7A8* and the immune microenvironment can lead to a breakthrough in the future treatment of LUAD.

Cancer is a disease characterized by dysregulated cell proliferation [24]. The knockdown of *CYP2S1* effectively inhibited the migration, invasion, and proliferation of LUAD cells, while improving the OS of LUAD patients [25]. In this study, we found that the overexpression of *SLC7A8* also effectively inhibited the proliferation and migration of LUAD cells. Bioinformatic analysis showed that *SLC7A8* was closely associated with the cell cycle. Indeed, SLC7A8 inhibited the expression of the cell cycle-related proteins CDK1 and CCNB1. Therefore, *SLC7A8* could check the progression of LUAD and improve its prognosis by influencing the proliferation and differentiation of LUAD cells.

This study has a couple of limitations. Firstly, although the relevant pathways were bioinformatically analyzed and experimentally verified, they were not validated through animal experiments, and we were unable to collect clinical samples for validation, which would have made the results more reliable. Secondly, we did not analyze extensive datasets, nor did we conduct multicenter cohort studies, Subsequent researchers may conduct multicenter studies based on this research.

This study demonstrated that the expression of *SLC7A8* was attenuated in LUAD tissues. *SLC7A8* can serve as a good prognostic marker for LUAD, and its overexpression inhibited the migration, invasion, and proliferation of LUAD cells.

## MATERIALS AND METHODS

### Data of LUAD patients

*SLC7A8* expression data in LUAD tissues and normal lung tissues were downloaded from TCGA and Xena. TCGA included 594 samples and Xena included 862 samples from the Genotype-Tissue Expression and TCGA databases. The clinical pathological and prognostic survival data of 522 LUAD patients were also downloaded from TCGA.

### Identifying *SLC7A8* expression in LUAD

The expression levels of *SLC7A8* in normal lung tissues and LUAD tissues were determined by performing expression analysis on TPM data from TCGA and Xena. The expression of *SLC7A8* in human normal lung epithelial cell and LUAD were determined by Western blotting.

### Evaluating the clinical value of *SLC7A8* in LUAD

After the LUAD patients were divided into groups according to their clinical pathological features, the expression levels of *SLC7A8* in LUAD tissues were determined based on TPM data from TCGA. The clinical diagnostic value of *SLC7A8* in LUAD was analyzed using ROC curves. Kaplan–Meier analysis and Cox regression were used to assess the prognostic value of *SLC7A8* in LUAD, and prognostic nomograms were constructed based on it.

### Cell culture

The human lung epithelial cell line BEAS-2b was purchased from Abclonal (Abclonal, China). The LUAD cell lines A549 and H1299 were purchased from American Type Culture Collection (ATCC, China). BEAS-2b cells were cultured in DMEM (Gibco, Thermo Fisher Scientific, Inc., USA) with 10% FBS (Gibco, Thermo Fisher Scientific, USA) and 1% Penicillin-Streptomycin solution at 37°C with 5% CO_2_. The LUAD cell lines were cultivated in Roswell Park Memorial Institute-1640 medium at 37°C with 5% CO_2_.

### Plasmid transfection

The SLC7A8 expression plasmid and control vector were synthesized by RiboBio (China). A549 and H1299 cells were cultured in 6-well plates to about 80% confluence and transfected with 2 μg of plasmid using Lipofectamine^®^3000 (Thermo Fisher Scientific, USA) following standard protocols. After 6–8 h, the culture medium was replaced with a standard medium.

### Western blotting

Total protein was extracted from A549, H1299, and BEAS-2b cells using RIPA buffer (Servicebio, China) and quantified using a BCA kit (Servicebio, China). The proteins were resolved by SDS–PAGE (Servicebio, China), transferred to PVDF membranes (Millipore, USA), blocked with 5% milk powder, probed overnight at 4°C with anti-SLC7A8 (1:1000, Abclonal, China), anti-CCNB1 (1:1000, Abclonal, China), anti-CDK1 (1:1000, Abclonal, China), and anti-β-actin (1:1000, Proteintech, China), incubated at room temperature for 1 h with anti-Rabbit IgG (1:3000, Jackson, USA) and anti-Mouse IgG (1:3000, Jackson, USA) secondary antibodies, washed with Tris-buffered saline with Tween 20, and finally detected using an ECL kit (Beyotime, China).

### Cell proliferation

The proliferation of A549 and H1299 cells was assessed using the CCK-8 and colony formation assays [26, 27]. For the CCK-8 assay, the transfected cells were seeded in 96-well plates and incubated with 10 μL of CCK-8 solution for 2 h. The absorbance was measured at 450 nm using a microplate reader at 24, 48, 72, and 96 h. For the colony formation assay, the transfected cells were seeded in 6-well plates, incubated for 14 days, and fixed for 1 h. The colonies were then photographed and counted.

### Wound healing assay

Cells were seeded in 6-well plates and cultured for 12 h to reach 100% confluence. The “wound” was made by scratching the monolayer with a sterile 200-μL pipette tip. Photos were taken immediately (0 h) and 24 h later at 100× magnification. The healing rate (healing width at 0 h/healing width at 24 h) was calculated using ImageJ.

### Transwell assay

The migration and invasion of A549 and H1299 cells were assessed using the Transwell assay [28]. To assess cell migration, the cells were cultured to the logarithmic growth phase, washed once each with phosphate-buffered saline and serum-free medium, suspended in serum-free medium at a concentration of 1 × 10^5^/mL, and seeded in the upper chamber of a 24-well Transwell chamber (Biosciences, USA). The lower chamber contained 600 μL of Roswell Park Memorial Institute-1640 medium containing 20% fetal bovine serum. After incubating for 24 h at 37°C, the lower chamber was fixed in 70% methanol for 1 h and stained with crystal violet. The cells in the low chamber were photographed and calculated. The protocol of the invasion assay was the same as that of the migration assay, except for the application of 100 μL of Matrigel to the upper surface of the membrane.

### Roles, mechanisms, and PPI network of *SLC7A8*-related genes

Correlation analysis identified 679 genes coexpressed with *SLC7A8* in LUAD tissues. The coexpression was considered strong when the correlation value r >0.3 or r <−0.3. The functions and mechanisms of the coexpressed genes were explored using Gene Ontology and Kyoto Encyclopedia of Genes and Genomes enrichment analysis, with *P* < 0.05 being the threshold for significance. The STRING database was used to construct a PPI network composed of the co-expressed genes, and Cytoscape was used for visualization. Based on the median value of *SLC7A8* expression, 535 LUAD cases from TCGA were sequenced and classified into two groups. GSEA was used to analyze the mechanisms of *SLC7A8* in LUAD progression.

### Correlation between *SLC7A8* expression and immune infiltration in LUAD

LUAD tissues from TCGA were evaluated using single-sample GSEA, and the relationships between *SLC7A8* expression and immune cell infiltration were analyzed using Spearman’s correlation. Moreover, this method was applied to explore the relationships between *SLC7A8* expression and immune factors.

### Statistical analysis

*SLC7A8* expression in LUAD tissues was investigated using the *t*-test [29]. The diagnostic value of *SLC7A8* in LUAD was analyzed by computing the area under the ROC curves. The prognostic value of *SLC7A8* was ascertained using Cox regression and Kaplan–Meier survival analysis. The survival curves were constructed using the median grouping method, where 50% of patients were defined as having high expression of SLC7A8 [30]. Correlation analysis was used to investigate the relationship between *SLC7A8* expression and immune cell infiltration in LUAD. *P* < 0.05 was considered to be significant.

### Availability of data and materials

The datasets used during the current study are available from the corresponding authors upon reasonable request.
